# Recombinant antibody production evolves into multiple options aimed at yielding reagents suitable for application-specific needs

**DOI:** 10.1186/s12934-015-0320-7

**Published:** 2015-09-02

**Authors:** Ario de Marco

**Affiliations:** Department of Biomedical Sciences and Engineering, University of Nova Gorica, Glavni Trg 9, 5261 Vipava, Slovenia

**Keywords:** Antibody fragments, Bacterial antibody display, Antibody-based fusion proteins, Neutralizing antibodies, scFv, VHH

## Abstract

**Background:**

Antibodies have been a pillar of basic research, while their relevance in clinical diagnostics and therapy is constantly growing. Consequently, the production of both conventional and fragment antibodies constantly faces more demanding challenges for the improvement of their quantity and quality. The answer to such an increasing need has been the development of a wide array of formats and alternative production platforms. This review offers a critical comparison and evaluation of the different options to help the researchers interested in expressing recombinant antibodies in their choice.

**Results:**

Rather than the compilation of an exhaustive list of the recent publications in the field, this review intendeds to analyze the development of the most innovative or fast-growing strategies. These have been illustrated with some significant examples and, when possible, compared with the existing alternatives. Space has also been given to those solutions that might represent interesting opportunities or that investigate critical aspects of the production optimization but for which the available data as yet do not allow for a definitive judgment.

**Conclusions:**

The take-home message is that there is a clear process of progressive diversification concerning the antibody expression platforms and an effort to yield directly application-adapted immune-reagents rather than generic naked antibodies that need further in vitro modification steps before becoming usable.

## Background

The worldwide therapeutic antibody market is estimated as over $50b/year, diagnostic market over $10b/year, and research market accounts for $3b/year, whereas the average annual growth rate for all antibody applications in the last 15 years is over 5 % and the recombinant antibody sector is one of the fastest growing. These numbers explain clearly the interest in all the biotechnological innovations aimed at improving the antibody production process in terms of absolute yields, structural stability and functional reliability of the final product. Specifically, the increasing attention dedicated to recombinant antibodies is due to the possibility of: (1) performing straightforward engineering by means of simple molecular biology techniques; (2) developing binders of variable formats and fused to different effectors and tags; (3) producing the final constructs inexpensively in microbial factories; (4) maintaining stable material clonality, the mandatory pre-requisite for result reproducibility [1]. This trend will probably accelerate in the foreseeable future because of the demanding structural features of the emerging antibody-based reagents such as Immunotoxins, Antibody Drug Conjugates (ADCs), Bispecific Antibodies, and Bispecific T cell Engager (BiTE) [2, 3]. Clearly, the great structural variability of the designed immune-reagents has its reason in the necessity to obtain effective reagents that are possibly also simple to use. At the same time, it implies the necessity of developing customized expression and purification procedures adapted to the construct specificities in order to assure sufficient yields. Such a complex context requires the capacity of comparing advantages and shortcomings of alternative platforms. This review aims at summarizing the recent trends, tries to evaluate the level of feasibility and reliability reached by the different methodologies, and will describe some recent innovative proposals.

## Production of antibody fragments in prokaryotic systems

### The conventional approach: expression in *Escherichia coli* (*E. coli*)

**Fig. 1:**
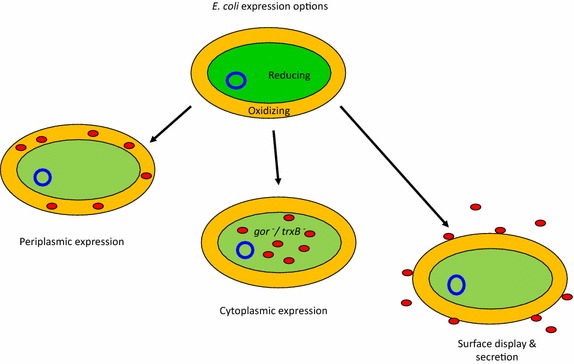
Conventional antibody expression in *E. coli.* Most of the recombinant antibodies rely on the formation of disulfide bonds in order to reach their native structure. Periplasm is the only *E. coli* oxidizing compartment compatible with disulfide bond formation and consequently it has been considered the logic environment for antibody accumulation despite its small volume. Mutant strains (*gor* ^−^/*trxB* ^−^) with partially oxidizing cytoplasm represent an alternative (cytoplasmic accumulation) as well as expression systems that enable the antibody secretion in the medium or at the cell surface (antibody display)

*Escherichia coli* still remains the most popular organism for recombinant protein expression and it still represents the standard for the production of antibody fragments on a lab scale. There are objective advantages for this choice, such as its simplicity, the availability of a large amount of well-tested reagents (vectors, strains), and the enormous experience (protocols, common expertise) that the research community accumulated over the last 30 years using this specific bacterium. The negative side effects implicit in choosing *E. coli*-based expression systems are that this solution is often driven by consuetude rather than by rational considerations. This means that both innovative approaches that exploit *E. coli* and systems based on other organisms might be neglected despite their use do not always requires further competences or particular equipment. In the case of antibody fragments, the conventional approach is to express them as secreted constructs that accumulate in the *E. coli* periplasm because this is the sole bacterial compartment that provides the oxidizing conditions and the combination of specific chaperone and isomerase activities necessary for the formation of correct and stabilizing disulfide bonds (Fig. 1). This approach enables the production of large amounts of functional antibody fragments and also their fusions to large proteins such as alkaline phosphatase and rhizavidin [4, 5]. However, in some cases the yields can be very low due to impaired secretion in the presence of too elevated expression levels and because of limiting chaperone availability in the periplasm [6–8]. Oxidizing conditions can be reproduced in the cytoplasm of mutant strains (Fig. 1) in which the thioredoxin and glutathione reducing pathways are blocked (such as Origami and derivatives), whereas the cytoplasmic overexpression of the periplasmic DsbC isomerase (such as in Shuffle T7 Express) can rearrange disulfide bonds involving incorrect cysteine residues. However, the published results obtained with these strains are contradictory [9, 10]. Although some groups claim successful production of functional antibody fragments in their cytoplasm, when critically evaluated, the results often indicate very low yields, while the biophysical characterization necessary to assess protein quality might be insufficient [11, 12]. In another case, the fusions between an anti-HER2 scFv and monomeric fluorescent proteins expressed in *gor*^−^*/trxB*^−^ strain seem to be functional [13] but the SPR data are not compatible with a 1:1 binding and, consequently, cast some doubts that the immune-reagents form (also? active?) aggregates. Some other positive reports should be considered carefully because of the chosen models. It is known that a significant portion of VHHs can accumulate as functional binders even in the absence of disulfide bonds and that consequently they can be expressed as functional intrabodies in the reducing cytoplasm of bacteria (and even of eukaryotic cells). When such structurally robust VHHs are expressed in Shuffle T7 Express bacteria, the resulting yields are very high. However, the production improvement with respect to the “control” BL21(DE3) bacteria is negligible [14]. Unluckily, no disulfide-dependent nanobody has been tested for comparison in such an experiment to evaluate the strain effectiveness under more demanding conditions.

**Fig. 2:**
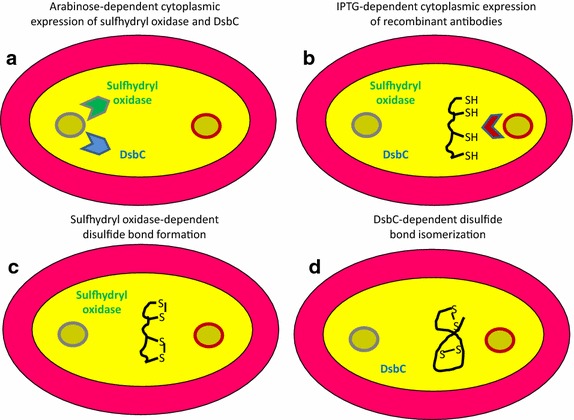
Alternative antibody expression in *E. coli* cytoplasm. Effective accumulation of functional recombinant antibodies can be obtained by expressing sulfhydryl oxidase and DsbC isomerase (**a**) in the cytoplasm before inducing antibody expression in the same cell compartment (**b**). The two foldases have complimentary activities: the cysteine SH groups are converted into disulfide bonds by sulfhydryl oxidase (**c**) and, if necessary, these are scrambled by DsbC to achieve the native folding (**d**)

Recently, antibody fragments have been produced very efficiently in the cytoplasm of *E. coli* overexpressing sulfhydryl oxidase (SO) and DsbC [15, 16] (Fig. 2). The method seems suitable for both VHHs and scFvs but its major advantage is that it enables the accumulation of functional immune-reagents with extremely more complex structural needs. It is the case of reconstituted IgG-like macromolecules as well as of fusions between an antibody fragment and proteins that have different redox requirements to fold correctly. The approach enabled the yield of tens of mg of monodispersed IgG/L using Luria–Bertani (LB) bacterial medium without any attempt of culture optimization. When conventional (periplasmic) and SO/DsbC cytoplasmic cultures were compared using the same constructs, the cytoplasmic yields were always significantly (at least 10 times) higher. The immune-reagents accumulated in the cytoplasm had also superior specific functional activity, indicating that folding was correct even in the case of complex structures such as IgG-like antibodies. This accomplishment appears as a clear improvement in comparison to the initial reports, which indicated the feasibility of using *E. coli* for IgG production [17, 18].

Expression rate control is a key parameter in recombinant production [6] and alternatives to the classical method based on *lac* promoter and its derivatives can contribute to higher antibody fragment yields. In the case of the pair formed by the *Pm* promoter and the benzoic acid-inducible XylS transcription activator [19], the direct evolution of XylS resulted in mutants that enabled ninefold higher yields of the tested scFv-phOx construct. Unfortunately, the paper does not report data describing the structural and functional features of the (over)-produced immune-reagent.

The strategies used for increasing the solubility of recombinant proteins expressed in *E. coli* have prevalently focused on conditions known for improving folding efficiency. Proteins such as isomerases and chaperones have been overexpressed, the bacterial growth has been performed at low temperatures and in the presence of osmolytes and alcohols known for stimulating heat-shock response, and even specific strains have been developed [7–9, 20]. By contrast, the optimization of medium composition and fermentation conditions is often neglected in academia with the exception of labs performing more industrial-oriented R&D. However, significantly higher amounts of antibody fragments can be recovered when the physical–chemical culture conditions are improved, and this is possible even in the absence of sophisticated fermentation equipment. Cell density can be increased tenfold in standard Erlenmeyer flasks when bacteria are grown using rich medium instead of LB [14] and the yields can be further improved by exploiting LEX™ bioreactors [21] or baffled flasks in combination with optimized shaking velocity [22].

**Fig. 3:**
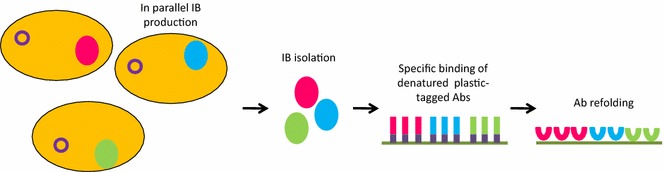
Surface–activated refolding of recombinant antibodies. Bacteria are first forced to produce inclusion bodies formed by recombinant antibodies fused to peptides affine for plastic. Once denatured, each single polypeptide adhere separately onto plastic plate surface by means of the peptide tag. The denatured polypeptides efficiently refold (solid-phase refolding) into functional antibodies because the unproductive interactions involving hydrophobic patches present at the surface of different folding intermediates are prevented

A completely different approach considers that functional recombinant antibodies can be obtained also by promoting first their accumulation in the inclusion bodies and then their refolding [23]. The idea is not new, but its implementation was significantly improved by the particularly effective strategy recently proposed by Kumada et al. [24]. These authors designed a high-density microplate culture system for producing in parallel insoluble constructs of scFv fused with polystyrene binding peptides at the concentration of 1 mg/mL and recovered functional antibodies by solid-phase refolding (Fig. 3). The efficiency of the approach is probably due to the fact that, after inclusion body denaturation, the single polypeptides adhere separately onto plastic plate surfaces by means of the polystyrene-affinity tag. This physical segregation reduces unproductive interactions among folding intermediates that can still expose hydrophobic patches. The method was validated successively with Fab fragments and further developed using alternative tags suitable for alternative refolding protocols [25, 26]. Its limit is that refolding efficiency is very sequence-dependent.

In a recent contribution [27], the yields of two constructs formed of the same scFv fused to the sequences corresponding to a toxin of either bacterial (*Pseudomonas* exotoxin A) or plant (saporin) origin were compared in *E. coli* and *P. pastoris*. Exotoxin A-scFv was apparently expressed better in bacteria, whereas saporin-scFv production was more successful in yeast. The authors’ conclusions were that the toxin origin strongly influences the production of functional immune-reagents in evolutionary related or distant organisms. This would be an interesting hypothesis to assess thoroughly. However, the experimental data are difficult to interpret since the immune-toxins accumulated as inclusion bodies when expressed in bacteria and the reported yields correspond to the amounts of soluble protein after refolding. Consequently, the variable yields could be due to the differential refolding efficiency rather than to specificities of the microbial factories. Furthermore, crucial experimental characterizations and descriptions necessary to evaluate the structural features of the immune-toxins are missing thus rendering final judgment impossible.

A very interesting development is represented by the attempt of obtaining homogeneous *N*-glycosylated recombinant antibodies in *E. coli* with the long-term aim of recovering the effector functions and tissue targeting specificity proper of IgG molecules [28]. The authors demonstrated that it is possible to achieve a homogenously glycosylated and degradation-resistant scFv by designing a glycosylation site in the linker region and using an *E. coli* strain in which the *N*-glycosylation machinery of the ε-proteobacterium *Campylobacter jejuni* has been transferred [28]. The post-transcriptional modification significantly improved the scFv stability and solubility, while did not affect its binding capacity because glycosylation has a distal location with respect to the antibody paratope.

*Escherichia coli* extract has been successfully used also for the cell-free production of “knobs-into-holes” bispecific BiTE IgG antibodies [29]. The knobs-into-holes technology enables the pairing of complimentary antibody arms but the efficiency of the IgG reconstitution is low when the two complimentary molecules are co-expressed in cells due to the usual variable accumulation rate of the two constructs. The cell-free approach overcomes this major drawback and results in high yields (several hundred mg/L) of functional antibodies that can be further improved by optimizing the reagent composition [30].

### Alternative bacteria suitable for specific applications

**Fig. 4:**
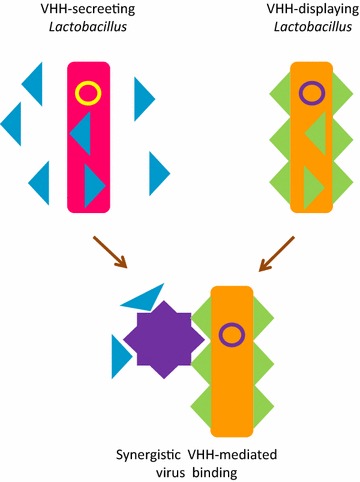
*Lactobacilli* as antibody-based functional food. *Lactobacilli* are transformed with expression vectors allowing for both recombinant antibody display and secretion to the medium. Such bacteria (lactobodies) maintain the capacity to produce recombinant antibodies after ingestion and promote the accumulation of virus-neutralizing binders in the midgut of the fed animals

*Lactobacillus* was the first alternative to *E. coli* to be proposed with the aim of providing biosafe bacteria expressing anti-viral nanobodies (lactobodies) for oral treatments in mammalians [31] and optimized strains are now available [32]. The concept considers the isolation of neutralizing VHHs and their expression as either secreted or cell-wall-anchored antibodies. Animals fed with such recombinant bacteria resulted better protected against virus-induced gastroenteritis since the nanobodies were successfully delivered in situ by the lactobodies. Lately, it has been demonstrated that VHH dimers targeting two independent rotavirus epitopes and combinations of soluble and displayed nanobodies blocking the same epitopes could further improve the treatment efficacy [33, 34] (Fig. 4). A similar philosophy led to the design of a system for the production of neutralizing scFvs in *Bifidobacterium longum* to use as a probiotic aliment [35]. The actual effectiveness of this approach was not demonstrated in vivo, but recombinant bacteria were able to express functional antibodies in the gut after intragastric administration, although the secretion efficiency apparently varied according to the antibody sequence. Although very promising as a vaccine complementary treatment, the implementation of this approach into approved cure for animal and human diseases [36] will probably suffer from the general public refusal of exploiting recombinant organism technology. Or, maybe, its simplicity will convince of the contrary.

Exotic bacteria such as the psychrophilic *Pseudoalteromonas haloplanktis* TAC125 have been proposed as an alternative to *E. coli* for VHH and scFv production [37, 38]. Although the choice of a microbial cell factory working at low temperature is interesting because it might favor the proper folding of the recombinant proteins, the practical advantages of this approach remain elusive, whereas some drawbacks should be considered, e.g. higher energy costs for bacterial culture, limited availability of expression vectors with application-friendly tags. Much more convincing are the data published recently by Mizukami et al. [39] that illustrate the enormous potential of *Brevibacillus choshinensis* for expressing recombinant antibody fragments. Two VHHs were expressed using a secretion vector and accumulated in the culture medium at concentrations of hundreds of mg/L when grown in flasks and topped at 3 g/L when produced in fermenter. The extremely accurate biophysical characterization of the resulting nanobodies confirms that they were functional and unquestionably monodispersed. It will be very interesting to follow the development of this production platform based on the “*Brevibacillus* in vivo cloning (BIC)” technology [40, 41] to understand if the excellent quantitative and qualitative features obtained with these model VHHs will be confirmed by using nanobodies with variable propensity to aggregation. It seems that larger/more complex proteins such as scFvs cannot benefit as much as VHHs [40, 41] from this expression methodology. At the moment it is difficult to evaluate whether the observed limitation is intrinsic of the organism folding machinery or of still-to-be-optimized expression vectors and fermentation conditions. The available information is not sufficient to evaluate the possible advantages of expressing recombinant antibody fragments in *Bacillus megaterium* and *Corynebacterium glutamicum* [42, 43]. Also *Pseudomonas putida* KT2440 does not seem to be a superior alternative to *E. coli* in terms of productivity, but at least this strain already obtained the biosafety certificate [44] and this may constitute a critical advantage for the preparation of therapeutic antibody fragments.

### Simplified approaches: the opportunities offered by cell display

**Fig. 5:**
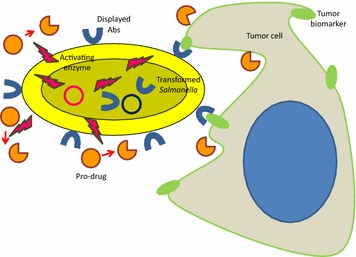
*Salmonella* as a tumor-targetable cargo. *Salmonella* can be transformed for displaying recombinant antibodies specific for tumor cell antigens and for secreting enzymes able to activate pro-drugs. The antibodies assure the selective accumulation of systematically provided bacteria in the antigen-expressing target tissue and there the secreted enzymes will trigger the local conversion of inactive pro-drug into toxic compounds with anti-tumor activity

Antibodies are too often considered as reagents that must be first expressed and purified before being used. Further biotechnological steps such as their functionalization with appropriate molecules may be necessary to enable their final application, as for instance their use as capture binders on chip surfaces or their complexation to nanoparticles. However, there are examples—as in cases mentioned above [31, 33, 35]—showing that some of these tedious and expensive steps can be avoided by expressing recombinant antibodies as displayed reagents exposed on the surface of bacteria. The therapeutic application of attenuated strains of VHH-displaying *Salmonellatyphimurium* has been demonstrated recently [45]. The rationale behind the experiment is that several bacterial strains can contribute to tumor eradication in mouse models because they may induce anti-tumor immune-response after direct local injection. Bacteria show also a certain natural tropism for tumors but it is insufficient for effective targeted accumulation after systemic treatment. To improve their tumor-delivery specificity, *Salmonella* has been engineered for displaying an anti-CD20 nanobody. Such engineered bacteria accumulated selectively in CD20-positive tumor xenografts when applied systematically in mice and were successfully used as a cargo to deliver an enzyme able to convert in situ pro-drugs into active compounds (Fig. 5). The efficient nanobody-mediated bacterial targeting resulted in a significant tumor rejection in vivo even in immune-compromised models [45]. A similar approach has been applied also to *Lactobacillus plantarum* to exploit it as a cargo to deliver cDNA to mammalian cells [46]. Specifically, lactobacilli were transformed to display a scFv selective for the dendritic cell marker receptor DEC-205. The resulting lactobacilli were effectively internalized in vitro and in vivo by dendritic cells and transferred efficiently a GFP plasmid to them.

**Fig. 6:**
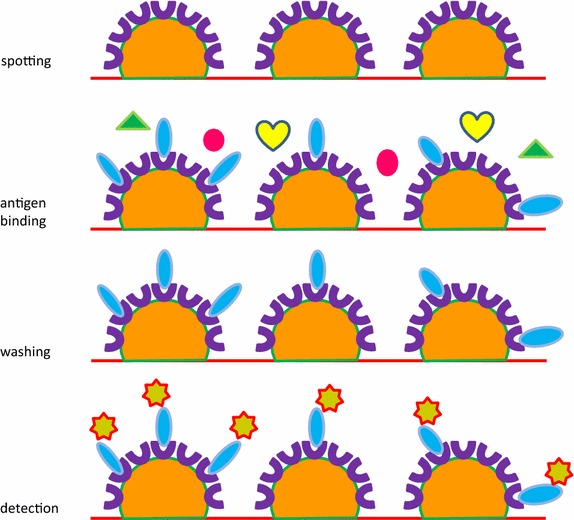
Purification-independent antibody-antigen recognition. The recombinant antibody expression is first induced in bacteria transformed with a membrane display vector. As a consequence, in few hours outward-oriented antibodies accumulate at the bacterial surface. The antibody-displaying bacteria are directly spotted on a chip surface. The displayed antibodies specifically capture the corresponding antigens that are labeled and quantified after a washing step necessary to remove the contaminants

In a diagnostic context, whole *E. coli* displaying anti-nucleophosmin VHHs resulted very effective when exploited as an antigen-capture reagent to bind the soluble antigen on microarray surfaces [47]. Contrary to the conventional approach used for biosensor surface immune-functionalization that relies on the availability of purified antibodies, bacteria were first transformed with a vector enabling the display of anti-target VHHs, the nanobody recombinant expression was induced for few hours, and finally the bacteria were directly spotted on the microarray surface. The high density of nanobodies inserted outwards on the bacterial membrane (Fig. 6) enabled an extremely high and selective binding of the corresponding antigens with signal-to-noise values superior to 10^3^ [47].

## Eukaryotic expression systems for antibody fragments

### Yeasts as microbial cell factories

The idea of displaying antibodies on whole cells has been long used in yeast for panning antibody libraries but only recently it has been proposed to exploit antibody-displaying whole cells as ready-to-use reagents. Binders were first selected from a scFv pre-immune library in yeast display format and the recovered cells were directly lyophilized for storage [48]. They retained their initial activity and specificity and could be used for few weeks representing a cheap and fast alternative to purified antibodies for at least some immune-capture applications such as the identification of soluble and membrane-bound pathogen antigens.

*Pichia pastoris* is the workhorse yeast for conventional production of scFv-derived antibody fragments. The protocols for obtaining secreted constructs are standardized and efficient [49–51] and most of the lately proposed improvements concern incremental features such as the length of the pre-induction glycerol feeding during fermentation, the codon usage, the optimization of the starting culture temperature, of the methanol concentration, and of pH obtained by means of Design of Experiment approach [52–54].

**Fig. 7:**
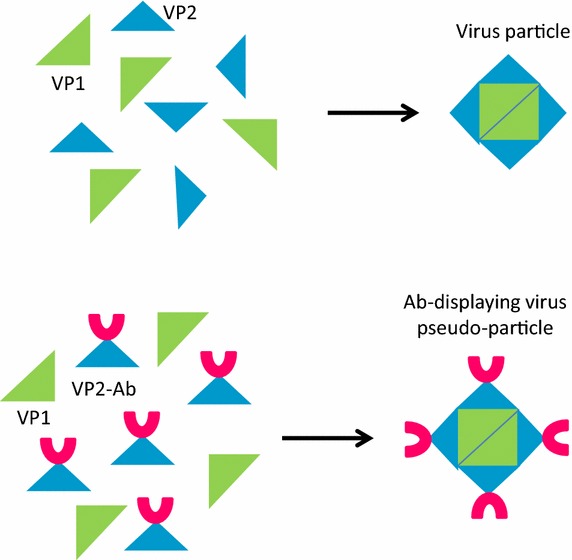
Multivalent antibody nanoparticles. Virus proteins can assemble into virus-like particles also when one of them is fused to antibody fragments. The outcome is represented by multivalent nanostructures exposing active antibodies. The nanostructure high avidity confers elevated neutralizing effect

Other yeasts are used as well for producing recombinant antibodies. In *Saccaromyces cerevisiae*, significant production increases (>100 folds) were obtained by a combination of strain engineering and the optimization of leader peptides for improving the secretion of full-length recombinant IgG molecules [55], even though the absolute yields remained modest (in the range of 10 mg/L). The same yeast was exploited for producing pseudo-type virus-like particles that form when scFv-Fc constructs fused to hamster polyomavirus-derived VP2 protein are co-expressed with the complimentary VP1 protein (Fig. 7). Such structures represent multivalent antibody nanoparticles possessing strong antigen-binding and -neutralizing activities that effectively prevented mammalian cell lysis induced by vaginolysin [56]. Another approach considers the *Saccaromyces* display of scFvs fused to non-self-cleaving Mxe GyrA intein for promoting the chemical functionalization of the antibody fragment by exploiting the Expressed Protein Ligation methodology. First, a thiol-nucleophile is used to release the antibody fragment from the yeast surface. As a consequence, the antibody acquires a thioester group at the carboxy-terminal that becomes available for binding covalently any molecule with an amino-terminal cysteine [57]. In such a way, the authors incorporated azido groups and biotinylated peptides into an anti-EGFR scFv. The production of antibody fragments (scFv-GFP fusion) in the fission yeast *Schizosaccharomyces pombe* seems possible [58] but does not exemplify an apparent advantage over other yeast- and prokaryotic-based systems.

Despite the fact that *P. pastoris* is one of the most established organisms for the expression of recombinant scFv and scFv-fusion proteins, only few reports describe the production of nanobodies in this yeast species. Since for many research labs yeast production might be technically more demanding than the golden standard *E. coli*, the choice would become meaningful only when specific advantages can be obtained. This evidence was missing in the first paper describing the production of a naked single-domain antibody in *P. pastoris*. Yields were comparable (10 mg/L) with those obtained with most of the VHHs expressed in bacteria [59]. The approach was more clearly justified in the work of Ji et al. [60] who used *Pichia* to express the IgG-like construct composed by an anti-TNF VHH fused with a human Fc. The authors found that it was more functionally active than the same construct expressed in the periplasm of *E. coli*. Nevertheless, the exact reasons of the functional advantage remain unknown since the authors did not characterize the recombinant antibodies for their structural features. In terms of yields, these were lower (5 mg/L) in comparison to what was obtained by Djender et al. [16] who expressed comparable constructs in bacterial cytoplasm. *S. cerevisiae* offers an interesting alternative to *Pichia,* but despite some promising initial results [61], it has never become a popular expression systems for VHHs. Recently, an important contribution assessed the role of specific residues in the VHH sequences the optimization of which might contribute substantially to improve their final yields in *S. cerevisiae* [62]. Specifically, the authors identified that some amino acids close to and inside the J segment are critical for VHH folding and secretion in yeast because they are involved in the chaperone recruitment in the ER. Their presence compensates for the recognition differences that exist between the mammalian and *S. cerevisiae* chaperone machineries and favor the VHH folding kinetics.

### Any good reason for expressing recombinant antibodies in mammalian cells?

Mammalian cells represent the gold standard for producing therapeutic antibodies but at the research lab level other systems have been preferred. However, the mammalian origin of VHH and scFv fragments suggests that it could be meaningful not only to produce them in yeast co-expressing mammalian chaperones [62], but directly in evolutionarily related organisms such as mammalian cells that should possess conserved secretion/folding machineries. The resulting antibodies are expected to gain the specific post-transcriptional modifications that enable the immune-activation and target recognition mechanisms in mammalians. This would represent a clear advantage for evaluating the in vivo biological activity of antibodies conceived for therapeutic applications. From this perspective, it would be very interesting to have access to comparative studies evaluating the binding characteristics, the biodistribution and pharmacokinetic, the immunogenic and curative effects, and the production costs of recombinant antibodies expressed in different organisms. However, sufficient information is not available so far with only few reports of antibody fragments produced in mammalians, such as a VHH against GFP expressed in CHO cells and scFv-Fc fragments against Epstein Barr virus in HEK293 [63, 64]. It is difficult to infer from this lack of data if the scarce interest finds its reasons in technical, biological or economic considerations.

### Filamentous fungi, insect-based systems and further “niche” eukaryotic organisms

*Trichoderma reesei* and *Aspergillus spp* failed to achieve the leading role in the production of recombinant nanobodies that was envisaged almost 15 years ago [61]. As suggested at that time, these expression systems should have been particularly effective for producing fusion proteins composed of antibody fragments linked to active enzymes such as oxidases and peroxidases that are structurally complex and consequently difficult to express correctly in bacteria. However, the recent report describing the expression in *Aspergillus* of a nanobody-glucoamylase fusion [65] does not provide sufficient information concerning yields and glycosylation patterns to evaluate the robustness of the method. Probably, the lack of a ready-to-use platform for cloning and expression discouraged many researchers to test filamentous fungi as a microbial cell factory for antibody fragment production. Another niche expression system that has been occasionally used to express recombinant antibody fragments and reconstituted IgG-like molecules is the *Leishmania tarentolae* eukaryotic parasite [66, 67]. The putative advantages of this method should reside in its codon usage but the reported yields are very low and probably do not justify the investment necessary to set this platform in a lab already equipped for recombinant production in another organism.

Insect cells and larvae have been proposed as nanobody expression living biofactories [68, 69]. Specifically, *Trichoplusia ni* larvae have been transformed by means of the efficient Improved Baculovirus Expression System to produce neutralizing nanobodies directed against diarrhea-inducing rotavirus A [69]. The characterization of the resulting VHHs indicated that they were functional but the shown data are too preliminary to assess the real benefit of this production approach in terms of simplicity, cost-efficiency, and antibody quality.

### Exploiting plants for antibody production

Recombinant antibody fragments have been successfully produced in different higher plants. For instance, an anti-murine TNF nanobody expressed in and purified from rice seeds was effective in neutralizing its antigen and suppressing the progression of collagen-induced arthritis in mice [70]. The claimed advantages of this approach with respect to microbial protein factories are low production costs, scalability, reduced safety issues such as toxin and virus contaminations, controlled *N*-glycosylation, and the possibility to concentrate the product in specific organs, usually seeds or leaves. Nevertheless, the final antibody concentration in plant organs is generally low (not exceeding hundreds of mg/kg plant, with antibodies representing usually less than 5 % of the total seed proteins and less than 1 % of leaf proteins). The downstream processing is the major system drawback since the diluted target product is mixed with a high load of soluble and particulate contaminants that require demanding integrated technological procedures for their removal [71]. Furthermore, recombinant protein production can induce physiological stress in the host plants resulting in the accumulation of heterogeneous products [72]. The involved mechanisms are not yet completely understood but recently it was demonstrated that the expression of VHH-Fc and scFv-Fc fusions trigger unfolded protein response in *Arabidopsis* seeds [73]. The most surprising result in this study was that neither the antibody expression levels nor the intrinsic structural stability of the constructs led to significant response differences. This observation suggests that the critical threshold for stress response is probably low, whereas other factors, such as the environmental conditions during seed development might contribute to final protein quality and yields. In general, the biophysical characterization of the antibodies expressed in plants have not been thoroughly addressed because the emphasis was rather placed on the (residual?) functionality of the purified material. Bivalent reconstituted Fc-VHH antibodies produced in *Arabidopsis* and *Nicotiana benthamiana* appeared partially degraded after the purification step but resulted effective in neutralizing avian influenza virus [74] and the α-cobratoxin venom [75]. Also in other cases the structural heterogeneity of antibodies produced in plants does not appear to compromise significantly their functionality [72, 76–78], but it can probably complicate their acceptance as therapeutics. Likely, the methodology still requires the optimization of the expression conditions and of the binder formats. Forcing the antibody fragment accumulation in specific sub-cellular compartments and the use of plant codons, specific promoters, fusion partners, and tags can increase the final yields of stable recombinant antibodies [79–82], although the optimal stabilizing combination seems to be rather antibody-specific, as exemplified in the case of the fusion of VHHs with different Fc domains [77].

Immunogenicity can be induced by proteins expressed in higher-plant due to the presence of some plant-specific sugar residues. The problem has been addressed by using RNA interfering technology to down-regulate glycosylation. A more elegant and definitive alternative was the engineering of *Nicotiana benthamiana* and of the moss *Physcomitrella patens* to obtain fully human *N*-glycosylation [83, 84]. Furthermore, such moss is suitable for photobioreactor production of antibody fragments [85] as well as of therapeutic IgG antibodies with superior ADCC (antibody-dependent cell-mediated cytotoxicity) compared to the same constructs recovered from mammalian cultures [86].

Also micro-algae can represent a valid antibody factory. The unicellular organism *Chlamydomonas reinhardtii* was able to express functional immunotoxins in both scFv- and IgG-like formats [87]. These large macromolecules have very complex structures that are difficult to recover correctly folded in most of the recombinant expression systems and this evidence can justify the interest in this protein factory.

A meaningful line of research is aimed at developing transgenic crops able to produce antifungal recombinant antibodies that neutralize pathogens in vivo. The approach is not absolutely new, but its efficacy strongly profited from the late technological progresses. By using such a strategy, it was possible to develop crops tolerant to different phytopathogen infections such as *Brassica napus* resistant to anti-*Sclerotinia sclerotiorum* scFvs [88], *Ciitrus* tolerant to *Citrus tristeza virus* [89], and soybean resistant to the *Fusarium virguliforme* toxin-1 that induces the sudden death syndrome [90].

### Neutralizing recombinant antibody fragment expression for direct therapeutic applications

In one of the previous sections we have seen how *Lactobacilli* overexpressing anti-viral antibody fragments can be directly used as an effective “therapeutic food”. It is possible because a relatively small volume of their culture delivers enough functional antibodies to mitigate the disease symptoms [31, 33]. Vegetal organisms can be exploited as factories for antibody production and for expressing those antibody fragments that can protect them from phytopathogens [88–90] but would a plant-based diet be able to provide therapeutic antibody doses sufficient to induce a beneficial effect in fed mammalians? There are promising results indicating the feasibility of such a smart development by which plant-producing recombinant antibodies might provide “functional food” with direct therapeutic effect. Transgenic rice has been engineered to accumulate neutralizing anti-rotavirus nanobodies in its seeds (MucoRiseARP1) [91]. The idea is that of protecting human populations against life-threatening diarrhea by feeding people with antibody-enriched seeds because rice is already part of their daily diet. Following the engineering of plants to suppress the upload of endogenous storage proteins, the nanobodies accumulated in the seeds at sufficiently high concentrations (g/kg of total weight and 11.9 % of total protein) to be effective in vivo as a prophylactic medicament. Furthermore, neutralizing nanobodies are water-soluble, resistant to both boiling and long-term storage (at least 1 year), and do not rely on cold-chain transport and storage. These features render the mutant rice not a simple basic research proof-of-principle but a product that could be potentially adopted by final users. In animal model the results seem very promising since even immunodeficient mice fed with MucoRiseArp1 resulted significantly protected against rotavirus infections. The strategy as well as the probable legal and cultural resistance is the same as described above for anti-virus neutralizing nanobodies expressed in *Lactobacillus*. It also reminds the logic that led to the development of the “Golden-rice” that was designed for providing a cheap source of beta-carotene to integrate the dietary Vitamin A shortage in rice-eating populations [92]. Hopefully they will not share the same tormented story. Another meaningful example of therapeutic plant is given by the transgenic peas accumulating anti-Eimeria neutralizing scFvs in their seeds. Chickens fed with them showed significant mitigation against the parasite-induced coccidiosis [93].

A different therapeutic approach considers the adenovirus vector-dependent expression of a hetero-trimer composed of VHHs specific for independent epitopes of both Stx1 and Stx2 Shiga toxins. The expression vector is delivered by a single intramuscular injection and the resulting VHH-based neutralizing agent is released to the circulation. As the consequence of effective and systemic toxin targeting, the methodology resulted highly protective against hemolytic-uremic syndrome in piglet model [94] and, as a vaccination method, could represent a valuable curative approach for a pathology for which no suitable remedy is available. Another peculiar exploitation of VHH in vivo expression has been proposed by De Vooght et al. [95] for impairing the tsetse fly-dependent spreading of trypanosome parasites. The authors demonstrated that it was possible to stably infect the fly population with the genetically engineered bacterial symbiont *Sodalis glossinidius* expressing a strong anti-trypanosoma nanobody. The nanobodies were efficiently secreted by transformed bacteria in vitro [96] and in vivo [95] and such bacteria were transmitted—although not very efficiently—in the progeny and promoted the accumulation of the trypanolytic nanobodies in several tissues among which the midgut that is an obligatory environment for the trypanosome development. The paper does not clearly demonstrate that is possible to select long-term paratransgenic tsetse populations in which the parasite transmission is impaired by the nanobody activity. Nevertheless, it evidences the drawbacks that must be removed to achieve this goal, such as the preventive elimination of the wild type bacterial population to avoid competition with the recombinant bacteria and the selection of nanobodies structurally resistant to the acidic and proteolytic conditions typical of midgut.

Another innovative development concerns the possibility to use plant-expressed antibodies for environmental remediation and by such a way preventing pollution conditions that are critical for human health. Specifically, Barbi et al. [97] developed transgenic tobacco expressing anti-microcystin-LR antibody fragments that are secreted and anchored to plasma membrane in leaf tissues. The membrane-retained antibodies were able to bind the microcystin initially present in hydroponic medium and formed stable complexes that could be eliminated removing the leaves.

### Recombinant antibody fragments as effective intrabodies

VHHs are often stable in the absence of disulfide bonds [98–100]. This characteristic makes them valuable tools for cell biology studies [101] as well as candidates for therapeutic applications based on their expression and accumulation as intrabodies directly in the cytoplasm/nucleus of the host cells. In this subcellular compartment they can neutralize the corresponding antigen and block its activity, as already demonstrated in the case of anti-viral VHHs [98, 99]. In the specific case of the anti-Rev VHH Nb190 that is able to suppress HIV-1 replication, cells stably expressing the intrabody were protected against the virus-induced cytopathogenic effect [102].

Different strategies have been developed in order to select directly for functional recombinant intrabodies. A C-terminal GFP protein allowed for the fluorescence-based light-microscopy screening of the clones that expressed folded nanobodies binding selected cellular structures [103]. However, the original method is demanding because the evaluation is made individually for each clone. Furthermore, the folding of several VHHs depended on DsbC isomerase and stabilizing tags that would not be available in clinical settings. Consequently, more convenient approaches have been considered and recently different variations of the two-hybrid technology were successfully applied for isolating both scFv and VHH intrabodies [100, 104–106]. Mukhtar and colleagues [104] successfully used the yeast two-hybrid technology to isolate human scFv intrabodies against nucleoproteins involved in influenza virus replication and transcription. Such intrabodies strongly bound their antigens and modified their cellular distribution and accumulation rate. When applied to porcine circovirus type 2-immunized VHH collections, yeast two-hybrid libraries enabled the isolation of intrabodies suitable for ELISA and immunocytochemistry on infected cells [105]. More recently, some VHHs targeting HIV-1 proteins have been isolated by Sos Recruitment System, a variation of the classical two-hybrid methodology in which the bait-prey interaction happens in the cytoplasm instead of the nucleus [100]. This alternative should prevent failures in all of the cases in which the folding efficiency of one of the two interacting polypeptides suffers from the conditions present in the nucleus milieu. However, only one of the selected candidates described in the paper was able to bind its viral antigen in the cytoplasm of eukaryotic cells leading to its delocalization and despite the strong binding the nanobody could not impair the cytostatic and apoptotic effects promoted by the antigen [100]. The results confirm that the approach still needs to be improved but can be suitable for identifying functional binders useful for studying the molecular mechanisms regulating pathogen proteins. On the other hand, they question the hypothesis of using antibodies for buffering pathogenic factors because incomplete neutralization could determine the approach failure, whereas antibody excessive accumulation could result toxic for the host cell. The problem could also lie in the antibody quality obtained by yeast selection methods since the low transfection efficiency of this organism limits the overall original clone variability. Therefore, transposing the two-hybrid principle into a bacterial system makes sense due to higher transformation efficiency i.e. a larger clonal diversity would become available for selection. The suitability of bacterial two-hybrid for the selection of VHHs was demonstrated by Pellis et al. [106] who succeeded in recovering functional intrabodies directed against GFP, HIV-1 integrase, and *T. vivax* nucleoside hydrolase. The nanobodies had stability and binding characteristics similar to those of VHHs isolated by phage display libraries constructed using the same RNA pool and, in some cases, the same clone was recovered by both selection methods. The authors underline how important was having used material from immunized animals since somatic maturation would have provided clones of sufficient binding affinity for obtaining effective two-hybrid coupling. However, most of the selected clones had affinity in the nanomolar range, namely binding constants that can be normally recovered panning sufficiently large pre-immune libraries [107]. Consequently, these should be considered also for this application. The results recently obtained using cytoplasmic Retained Display [108] confirm that pre-immune libraries are suitable for direct intrabody recovery, in this specific case scFvs with fixed human framework and CDR1/CDR2, and which can tolerate CDR3 diversification.

### Any help from in silico resources?

Antibody fragment final yields are clearly influenced by their propensity to misfold and aggregate. A first step for the selective screening of stable binders issued from phage display libraries can be achieved before starting the panning procedure by introducing a heating treatment of the phage population [109]. Since thermal stability and the capacity to refold into native structure inversely correlate with aggregation readiness, the heat-resistant clones are on average more stable than precipitated ones and can be purified by incubation at high temperatures [110]. Bioinformatics and protein modeling could further help in identifying critical residues able to improve the stability of the recombinant antibodies selected after panning. This approach should be considered, although it is difficult to identify a consensus about the hot spots even in the case of simple single-domain molecules such as the VH(H)s [111–113].

## Conclusions

Protein complexity implies the necessity of identifying customized answers to the variable problems related to their production. In the case of antibody fragments, the yields of single binders can change dramatically due to minimal sequence variations. Consequently, one cannot expect to obtain a single optimal universal production method, but rather should be aware of different options suitable for improving the final results. Many of the publications reviewed in this work share a common aim—the production of application-specific immune-reagents rather than naked antibodies to use indiscriminately—and complimentary strategies to accomplish it. Therefore, antibodies fragments are often already selected in vitro with the aim to recover clones possessing determined features such as epitope-specificity or neutralizing activity, then cloned as fusions with appropriate effector proteins, and finally diversified expression systems are chosen for yielding application-specific functional molecules (Table 1). The overall production design largely varies in terms of complexity but it must provide reagents suitable for the actual final needs. On the one extreme of the scale, simplified systems such as antibody displaying cells that can be used without any purification step are suitable for some simple immune-detection applications and their exploitation makes sense because of simplicity and low costs. On the other extreme, very sophisticated strategies have been successfully implemented for preventing pathogen aggression in vivo. In between, smart applications such as the development of immune-effectors and antibody-based functional food that work because the antibody fragments have been developed according to criteria that take carefully in account the in vivo physiological conditions in which they will operate.

**Table 1:** Recombinant antibody production: hot topics and examples

Antibody applications	Opportunity and challenges	Achievement reports (references)
Protein delivery	Fusions of Ab fragments with active proteins (IL1, toxins, chromophores,…)	[4, 5, 12, 16, 87]
BiTE	Bispecific T-cell engager	[29]
Bispecific activity	Possibility to interfere or to join two different pathways	[30]
Cell display	Purification-independent methods for inexpensive Ab production	[31, 33, 47, 48, 97]
Viral nanoparticles	Development of self-assembling multivalent structures	[56]
*In vivo* expression and activity	Production of pathogen-neutralizing Abs directly in food and animals	[88, 90, 91, 93, 95]
Immunomodulation	Effective glycosylation for precise targeting and CDC/ADCC	[28, 76, 85, 86]
Cargo delivery	Ab chemical functionalization for ADC, radiotherapy, and imaging	[57]
Bacterial delivery	Immune-response and accumulation of active molecules	[45, 46]
Intrabodies	Necessity to fold into active form in cell reducing cytoplasm	[100, 105, 106]

Recombinant antibodies are currently used in multiple applications which require the production of large amounts of qualitatively well-defined reagents that need as few as possible post-purification steps before being ready for the final application. Major biotechnological needs and representative examples of meaningful strategies for their accomplishment are listed below

Altogether, the picture looks optimistic because of the many published achievements. Nevertheless, the drawback of the present model for research production dissemination is its unbalance towards the exclusive publication of positive results. The consequence is that one has access to success stories but knows very little about conditions that resulted in failures and that would be useful to know in order to avoid similar mistakes. Systematic comparisons based on combinatorial approaches [114] and benchmarking initiatives [115, 116] are extremely useful to evaluate methods, experimental sets or reagents but are still rare in the field of recombinant antibody production [27, 60, 78, 86, 117]. Another observed limit is represented by the publication of papers in which controls and characterization data necessary to assess the reliability of the claimed conclusions are missing. For instance, data reporting yield improvement should be considered with some caution in the absence of control results showing that the antibody functionality is not affected by the quantitative increase since absolute and functional yields might be not coincident. Unluckily, the presentation of a complete set of control data is rare and it is a pity because potentially good ideas remain unfulfilled when reliable information is diluted in the presence of ambiguous results. Accordingly, methods presented without preliminary scientific reviewing, such as patent applications [118], are not discussed in this work. Probably, the community (authors, editors, funding agencies) should take more seriously the necessity to request minimal standards of biophysical [119] and functional characterization of immune-reagents and to reward the attempts to improve this praxis [120].

Summarizing and apart from “still-to-prove” options, the positive news is that new effective methodologies are available (Table 2). The actual limit resides in the fact that an up-to-date scientific work requires constantly increasing amount of skills (and sometimes new expensive equipment) for covering all the project steps optimally. The consequence is that in small labs some innovations might be slow to be implemented and some steps are consequently performed with sub-optimal techniques. As an example, the multifactorial statistical Design of Experiment methodology [121] would be beneficial in most of the works aimed at optimizing the production conditions and at characterizing the antibody binding features. However, despite the positive reports obtained also with antibodies [122–125], it is not largely used in academic labs because it requires a critical initial time investment for its setting. The same reasoning can be formulated for any specific expertise that participates to a project that—for instance—starts with the panning design to finish with a clinical application. The necessity for collaborations is evident but maybe the professionals of the recombinant protein (antibody) production should think about what competences would maximally qualify their contribution to projects that become more and more multidisciplinary and that should avoid bottlenecks at any step of their development. Finally, it can be difficult to switch from *E. coli* to—let say—the moss *Physcomitrella patens* or the microalgae *Chlamydomonas reinhardtii* for producing high-quality immune-reagents but why not to consider this strategy if, in the long term, it can result more convenient than idle time investment in a more familiar platform with clear intrinsic limits?

**Table 2:** Summary of the evaluated expression systems for recombinant antibody production

Expression organism	Method robustness: specificities	References (for unusual organisms)
*Escherichia coli*	Prokaryotic gold standard, different methodologies	–
*Lactobacillus paracasei*	Effective diet therapy	[31, 36]
*Lactobacillus rhamnosus*	Effective diet therapy	[32]
*Lactobacillus plantarum*	Effective cargo in vivo	[46]
*Bifidobacterium longum*	Diet therapy to be demonstrated	[35]
*Pseudoalteromonas haloplanktis*	Protein folding at low temperature: high energy needs?	[37, 38]
*Brevibacillus choshinensis*	Highly productive for VHHs	[39–41]
*Bacillus megaterium*	No shown advantage	[42]
*Corynebacterium glutamicum*	No shown advantage	[43]
*Pseudomonas putida*	FDA biosafety certificate	[44]
*Salmonella* *typhimurium*	Suitable for display and in vivo cargo applications, biosafe	[45]
*Pichia pastoris*	Eukaryotic gold standard	–
*Saccaromyces cerevisiae*	Suitable for engineering	–
*Schizosaccharomyces pombe*	No shown advantage	[58]
*CHO*	Industrial gold standard: no shown advantage at research lab level	[63]
*HEK293*	No shown advantage at research lab level	[64]
*Trichoderma reesei*	Not thoroughly characterized	[61]
*Aspergillus spp.*	Not thoroughly characterized	[61]
*Leishmania tarentolae*	Favorable codon usage, low yields	[66, 67]
*Trichoplusia ni*	Positive preliminary results	[69]
*Arabidopsis thaliana*	Product heterogeneity	[73, 74]
*Nicotiana benthamiana*	Product heterogeneity	[75]
*Oryza sativa*	Optimized yields and quality	[91]
*Physcomitrella patens*	Suitable for photoreactor, promising for IgGs	[84–86]
*Chlamydomonas reinhardtii*	Promising for large immune-reagents	[87]
